# Correlation between inflammatory markers over time and disease severity in status epilepticus: a preliminary study

**DOI:** 10.3389/fneur.2024.1334415

**Published:** 2024-02-02

**Authors:** Xiangsong Shi, Xiulin Zhang, Sumeng Song, Heyue Pan, Chengbing Huang, Taipeng Sun, Shouyong Wang, Jianyang Xu

**Affiliations:** ^1^Department of Neurology, Huai'an Third People's Hospital, Huai'an, China; ^2^Department of Psychiatry, Huai'an Third People's Hospital, Huai'an, China

**Keywords:** convulsive status epilepticus, inflammatory markers, NLR, PLR, SII, STESS

## Abstract

**Objectives:**

Convulsive status epilepticus (CSE) is a major subtype of status epilepticus that is known to be closely associated with systemic inflammation. Some important inflammatory biomarkers of this disorder include the neutrophil-to-lymphocyte ratio (NLR), platelet-to-lymphocyte ratio (PLR), monocyte-to-lymphocyte ratio (MLR), systemic immune inflammation index (SII), and pan-immune inflammation value (PIV). This study aimed to determine the NLR, PLR, MLR, SII, and PIV levels before and after treatment in adult patients with CSE and investigated the relationship of these parameters with disease severity.

**Methods:**

This retrospective study analyzed data from 103 adult patients with CSE and 103 healthy controls. The neutrophil, monocyte, platelet, and lymphocyte counts, as well as the NLR, PLR, MLR, SII, and PIV, were compared in adult patients with CSE during acute seizures (within 2 h of admission) and after treatment relief (1–2 weeks of complete seizure control). Furthermore, multivariate linear regression analysis investigated the relationship between NLR, PLR, MLR, SII, and PIV with the Status Epilepticus Severity Score (STESS).

**Results:**

The data revealed significant differences (*p* < 0.05) in neutrophils, monocytes, lymphocytes, NLR, PLR, MLR, SII, and PIV between adult patients with CSE during acute seizures and after treatment relief. The average neutrophil count was high during acute seizures in the patient group and decreased after remission. In contrast, the average lymphocyte count was lower after remission (*p* < 0.05). Furthermore, significant differences (*p* < 0.05) were observed in monocytes, lymphocytes, platelets, NLR, PLR, MLR, and PIV levels between adult patients with CSE after remission and the healthy control group. Multivariate linear regression analysis showed no significant correlation between NLR, PLR, MLR, SII, and PIV with STESS.

**Conclusion:**

The results of this study indicated that adult patients with CSE experienced a transient systemic inflammatory response during acute seizures, which gradually returned to baseline levels after remission. However, there was a lack of robust clinical evidence correlating the severity of adult CSE and systemic inflammatory response.

## 1 Introduction

Status epilepticus (SE) is an extremely common emergency in medicine. Rapid termination of clinical and electrical seizures is a key aspect of its treatment (1). The incidence of SE lies between 9.9 and 41 out of 100,000 individuals/year, exhibiting a bimodal distribution with peaks in individuals < 10 and > 50 years old (2). The International League Against Epilepsy (ILAE) proposed a new definition for SE in 2015. The revised definition applied to all seizure types, which explained SE diagnosis and established that long-term adverse events were dependent on the type and duration of seizures (3). Particularly, tonic–clonic SE has a duration of t1 (5 min) and t2 (30 min), while t1 (10 min) and t2 (60 min) were indicated for focal SE with altered consciousness. Convulsive status epilepticus (CSE) is more likely to result in complications than non-convulsive SE. The complications include cerebral edema, hypoxemia, aspiration pneumonia, electrolyte imbalances, arrhythmias, and rhabdomyolysis. The duration of seizures is substantially correlated with mortality rates in adult SE patients and increased medical costs (4). However, currently, there is a lack of established biological markers to facilitate an accurate assessment of the prognosis of status epilepticus. Although the Status Epilepticus Severity Score (STESS) is generally used to evaluate the severity and predict the prognosis of SE patients, clinical validation of the results is inconsistent (5, 6). Hence, it is of great significance to explore biological markers to accurately evaluate the prognosis risk of status epilepticus.

Novel systemic inflammation markers include neutrophil-to-lymphocyte ratio (NLR), platelet-to-lymphocyte ratio (PLR), and monocyte-to-lymphocyte ratio (MLR). These data are derived from routine blood tests, making them a rapid and cost-effective clinical application. Associations have been previously reported with elevated NLR, PLR, and MLR and central nervous system disorders such as neuromyelitis optica spectrum disorders and acute cerebrovascular diseases (7, 8). Additionally, novel systemic inflammation markers, such as the systemic immune inflammation index (SII) and the pan-immune-inflammation value (PIV) have been beneficial for the prognostic evaluation in cases, including ST-elevation myocardial infarction treated with stent implantation and obstructive colon cancer (9, 10).

Increasing evidence suggests that neuroinflammation and status epilepticus influence each other (11). In animal models, inflammation is both a cause and consequence of epilepsy (12). Moreover, in clinical studies, it has been confirmed that the white blood cell–endothelial cell adhesion mechanism plays a role in epileptic seizures (13). Recent literature has shown that neutrophils are involved in the inflammatory response before and after generalized tonic–clonic seizures. Furthermore, NLR is significantly higher in the acute phase of status epilepticus than in the subacute phase and control groups (14, 15). However, inflammatory markers in SE are rarely reported, such as SII and PIV. This study comprehensively compared changes in NLR, PLR, MLR, SII, and PIV before and after treatment of status epilepticus, analyzing their clinical potential based on the severity of the condition in conjunction with STESS.

## 2 Materials and methods

### 2.1 Study population

The clinical data from 165 patients diagnosed with SE and admitted at the Epilepsy Center of Huai’an Third People’s Hospital between January 2016 and January 2023 were retrospectively analyzed. The inclusion criteria included patients with CSE defined as the SE diagnostic criteria (3): (1) generalized tonic–clonic seizures >5 min or without recovery of consciousness between two episodes; (2) focal motor seizures with impaired consciousness > 10 min. Patients ≥ 16 years old were enrolled and their blood routine samples were collected within 2 h of admission and 1–2 weeks after achieving complete control of seizures through treatment. The exclusion criteria included the following: (1) non-CSE; (2) psychogenic non-epileptic seizures; (3) concomitant hematologic or autoimmune diseases; (4) using anti-inflammatory drugs or immunosuppressants in the past month; and (5) incomplete clinical data.

Of the 165 patients, 25 were <16 years of age, 8 patients had non-CSE, and 29 patients had incomplete full blood cell data. Finally, 103 patients were included in this study. The control group comprised 103 age- and gender-matched healthy individuals. They had no history of chronic disease and had not used anti-inflammatory drugs or immunosuppressants in the past month. The Ethics Committee of Huai’an Third People’s Hospital (Approval Number: 2023-010) approved this study.

### 2.2 Methods

The data included gender, age, seizure type, admission consciousness status, and STESS. BC-5390CRP automated hematology analyzer (Mindray Corporation) was used to collect complete blood counts. Blood routine data, including WBC, neutrophil, lymphocyte, monocyte, and platelet count, were collected during acute CSE (within 2 h of admission) and post-treatment relief (1–2 weeks of complete seizure control). The calculations for NLR, PLR, MLR, SII, and PIV were done based on the blood routine data (NLR = neutrophil count/lymphocyte count; PLR = platelet count/lymphocyte count; MLR = monocyte count/lymphocyte count; SII = platelet count x neutrophil count/lymphocyte count; PIV = neutrophil count x monocyte count x platelet count/lymphocyte count). Clinical physicians assessed the severity of patients with CSE using the STESS scoring system, ranging from 0 to 6, with higher scores indicating greater disease severity (16).

### 2.3 Statistical analysis

Statistical analysis was performed using SPSS 26.0 statistical software. Experimental data were expressed as means ± standard deviations; *t*-test and non-parametric tests were used to analyze normally distributed and non-normally distributed continuous data, respectively. The chi-square test analyzed categorical data, while multiple linear regression analysis was used to identify STESS-related risk factors. *p* > 0.05 indicated no statistical significance, while *p* < 0.05 indicated statistical significance.

## 3 Results

### 3.1 Baseline characteristics

The study population comprised 57 females (55.3%) and 46 males (44.7%). The average age of the 103 patients included in the study was 42.53 ± 16.66 years. Among them, 80 patients experienced tonic–clonic seizures (77.7%), and 23 had focal motor seizures with impaired consciousness (22.3%); 92 patients (89.3%) had a prior diagnosis of epilepsy upon admission; 90 patients (87.4%) had STESS scores of 0–2 points, while 13 patients (12.6%) scored >2 points. Demographic characteristics of the healthy control group are presented in Table 1. Table 2 shows that the STESS score was predominantly ≤2 with a statistically significant difference (*p* < 0.001) in patients with a history of epilepsy.

**Table 1:** Demographic and clinical characteristics of the two study groups.

	SE (*n* = 103)	Control group (*n* = 103)	*P*
Gender (M/F)	46/57	50/53	0.576
Age (year)	42.53 ± 16.66	42.65 ± 12.86	0.648
**State of consciousness (*n*, %)**			
Confusion of consciousness	72 (69.9%)		
Drowsiness	3 (2.9%)		
Trance	2 (1.9%)		
Stun	26 (25.3%)		
**SE classification (*n*, %)**			
Generalized convulsive SE	80 (77.7%)		
Focal motor SE	23 (22.3%)		
**Previous diagnosis epilepsy (*n*, %)**			
Yes	92 (89.3%)		
No	11 (10.7%)		
**STESS (*n*, %)**			
0–2	90 (87.4%)		
>2	13 (12.6%)		

**Table 2:** Fisher’s exact test between previous epilepsy history and STESS score ≤2.

Previous diagnosis epilepsy (*n*, %)	STESS ≤ 2 (*n* = 90)	STESS > 2 (*n* = 13)	X^2^	*p*
Yes (*n* = 92)	88	4	53.469	<0.001
No (*n* = 11)	2	9		

### 3.2 Laboratory testing

#### 3.2.1 Comparison of neutrophil and monocyte counts in adults with CSE during acute seizures and after treatment

**Figure 1:**
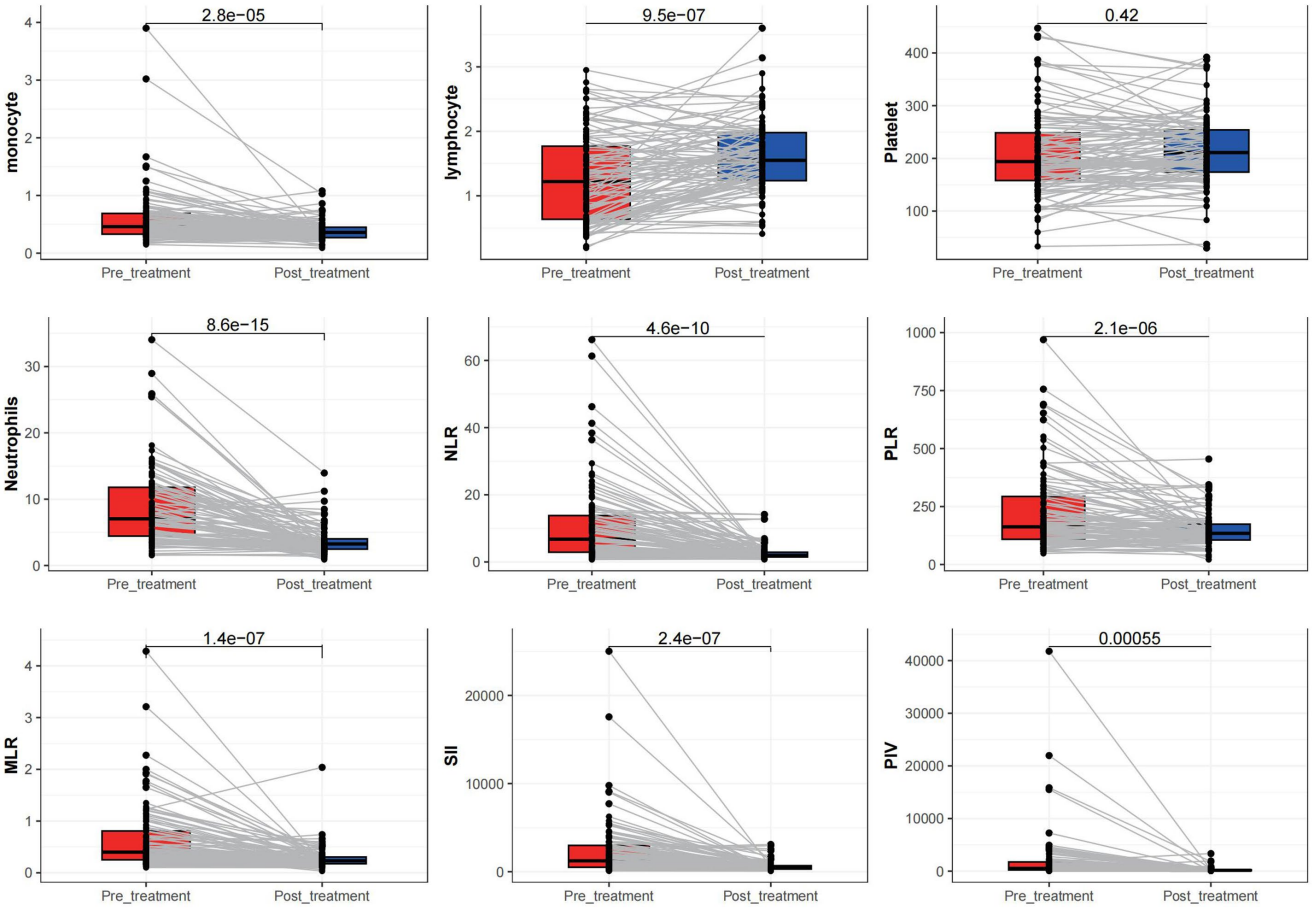
Comparison of different measured parameters between the patient groups at acute onset and after treatment remission.

A significant increase in neutrophil and monocyte counts and a significant decrease in lymphocyte count was observed in adults with CSE during acute seizures and after treatment (all *p* < 0.05). Furthermore, significant differences in NLR, PLR, MLR, SII, and PIV between CSEs and control groups were observed (*p* < 0.05). However, the platelet count difference was not statistically significant before and after treatment either in the CSEs (*p* > 0.05; Figure 1).

#### 3.2.2 Comparison between the adult CSE treatment relief group and the control group after treatment

**Figure 2:**
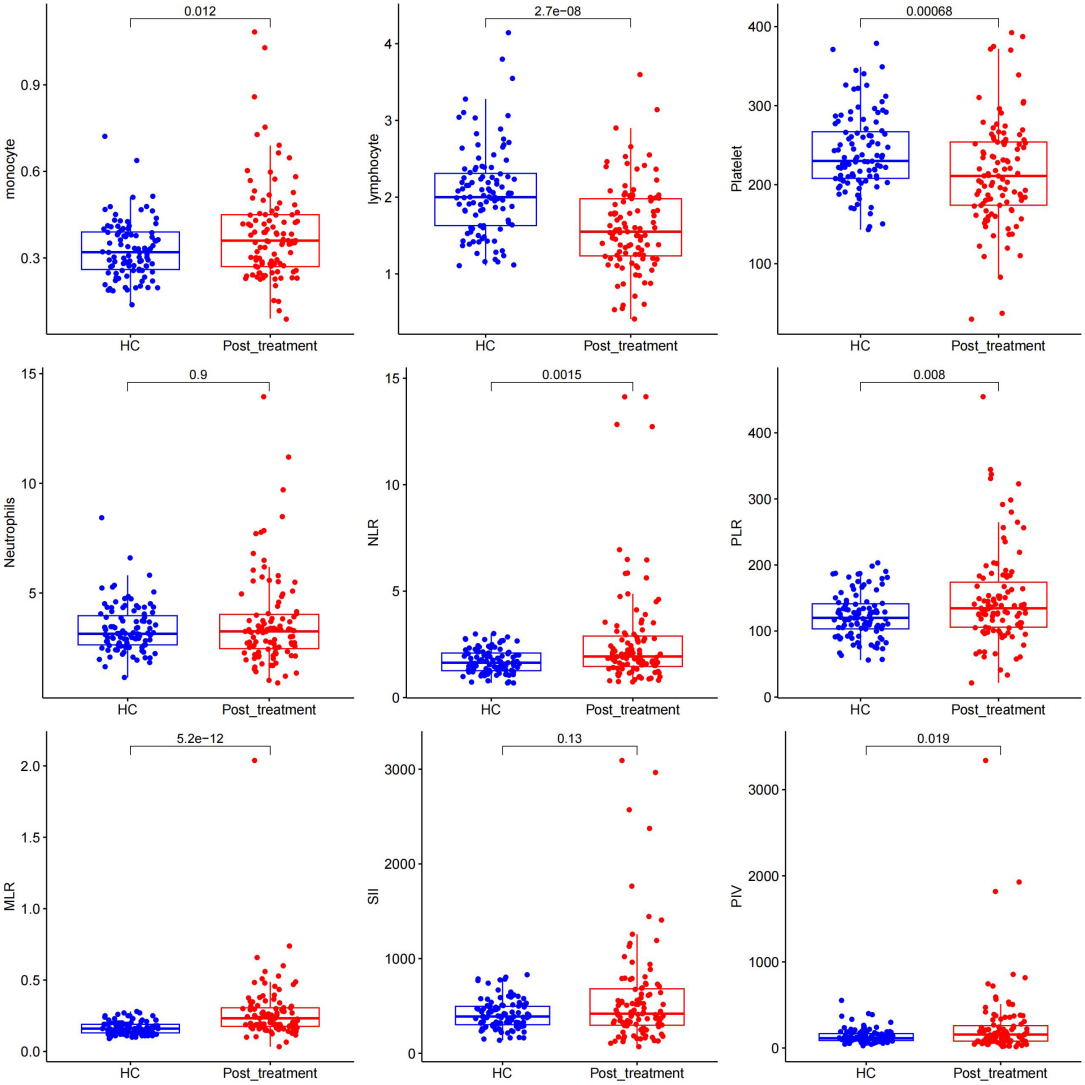
Comparison of different measured parameters between the patient group and the control group after treatment remission.

After relieving CSE in adults, the monocyte count was significantly higher in the treatment group than in the control group; however, lymphocyte and platelet counts in the CSE group were significantly lower (all *p* < 0.05) than in the control group. Moreover, the two groups had significant differences in NLR, PLR, MLR, and PIV (all *p* < 0.05). However, there was no statistically significant difference in the neutrophil count and SII between CSEs and control groups (*p* > 0.05; Figure 2).

### 3.3 Analysis of risk factors for the severity of status epilepticus in adult patients

**Figure 3:**
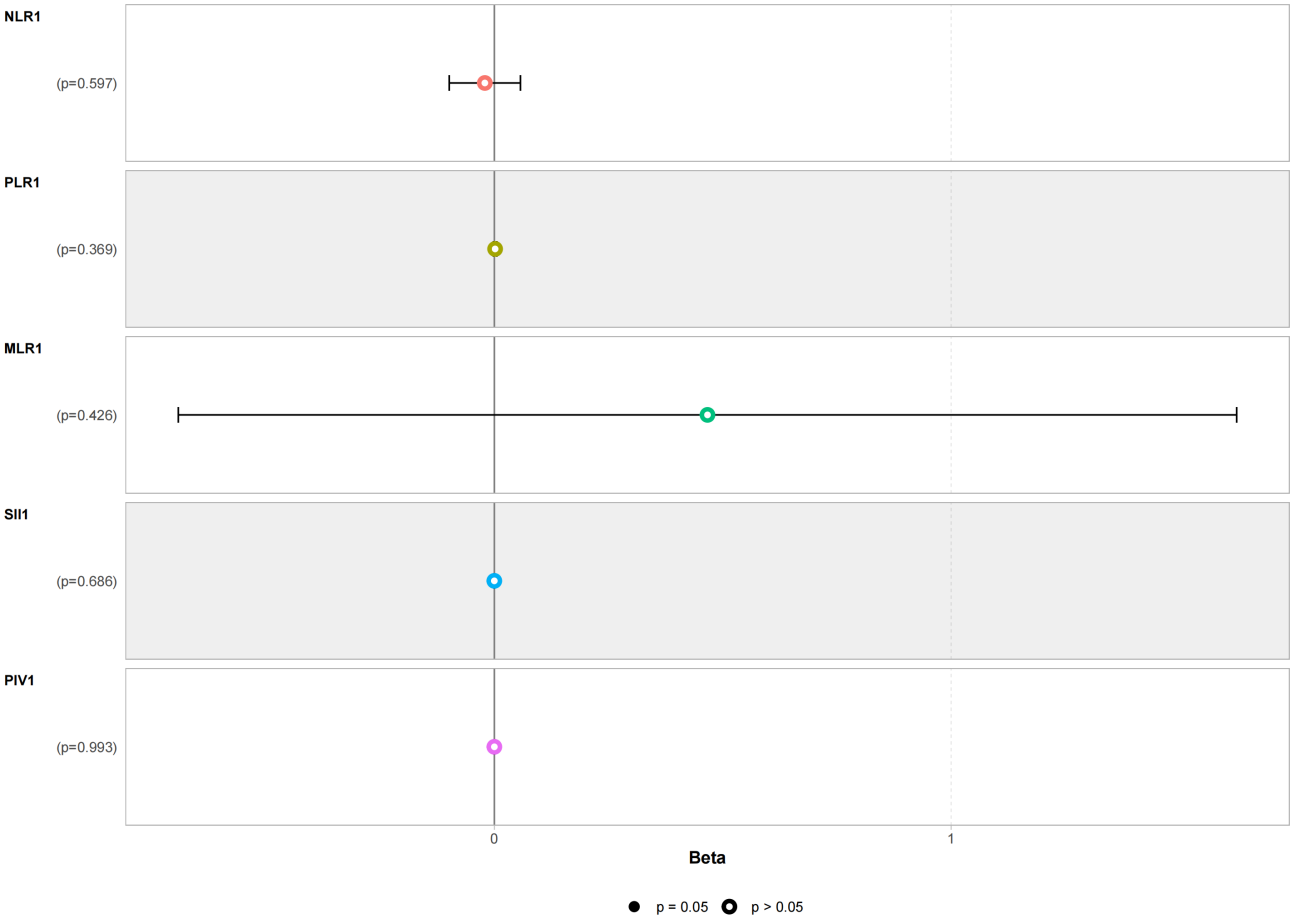
Multivariate linear regression analysis of the severity of status epilepticus in the patient group.

Multivariate linear regression analysis was conducted using NLR, PLR, MLR, SII, and PIV as independent variables and with STESS as the dependent variable. The multivariate linear regression analysis results revealed no significant correlation between NLR, PLR, MLR, SII, PIV, and the severity of the condition. Refer to Figure 3 for details.

## 4 Discussion

In this study, a comparison of the different parameters before and after treatment revealed that the acute phase of CSE elevated the neutrophil and monocyte levels and decreased lymphocytes, with no change in platelet counts. This suggests that only white blood cells may be involved in the seizure process. Interestingly, the transient increase in neutrophils and SII during the acute phase might indicate that neutrophil-mediated inflammatory responses are one of the mechanisms of CSE.

NLR has consistently performed well as a prognostic marker of various inflammation-related diseases, including epilepsy. However, novel markers such as SII have also demonstrated high predictive value (17–19). Status epilepticus, a special state of epilepsy, is associated with systemic inflammatory responses. It is believed that indicators like NLR and SII will be valuable and may even become independent predictive markers in diagnosing and predicting CSE. This study included patients who experienced CSE due to variations in NLR across different types of epileptic seizures. A prospective study reported that white blood cell counts increased during a single generalized tonic–clonic seizure, returning to baseline levels within 2 h (20). Moreover, white blood cells and neutrophil counts have been reported to increase significantly after generalized epileptic seizures, and inhibiting neutrophils could reduce seizure activity (21, 22). Simultaneously, a strong correlation between MLR, SII, and PIV inflammatory markers and status epilepticus was observed in adults. Tan et al. (23) reported that NLR and SII can differentiate status epilepticus from prolonged non-epileptic psychogenic seizures. On the contrary, other studies have found no significant correlation between SII and seizures associated with moderate-to-severe hypoxic–ischemic brain injury in infants (24). Status epilepticus is an extremely complex process that involves multiple factors, such as neuronal damage, inflammatory responses, and immune cell activation. Consequently, a single systemic inflammatory marker might not be suitable to explain the occurrence and persistence of status epilepticus. Therefore, a comprehensive assessment of multiple indicators such as NLR, PLR, MLR, SII, and PIV might provide a more accurate and objective evaluation.

STESS, developed by Rossetti et al. (16), is the first prognostic scoring tool for status epilepticus. It can be determined easily upon a patient’s admission and is widely used for grading the severity and outcome prediction of status epilepticus patients. Unfortunately, the results show no significant correlation between NLR, PLR, MLR, SII, PIV, and STESS. This may be due to the limitations of STESS in clinical use. Some studies suggest that STESS is only meaningful for predicting in-hospital mortality in patients with status epilepticus and is not useful for evaluating long-term prognosis (25). Etiology serves as an autonomous determinant of mortality in cases of status epilepticus, while the majority of patients presenting with status epilepticus cannot promptly ascertain the underlying cause upon admission to the medical facility (26). The patients included in this study were all effectively treated, possibly excluding those with poor treatment outcomes, which may have affected the effectiveness of STESS. Revisiting the clinical data of the study subjects revealed that the STESS scores of most patients with a history of epilepsy and status epilepticus were ≤2, indicating a favorable prognosis. Previous studies have reported that systemic immune response correlates to epilepsy severity and prognosis, but our results did not find a correlation between NLR, PLR, MLR, SII, PIV, and STESS scores. This negative finding lack of association could be related to our small sample size. We will increase the sample size to further clarify the reasons for this relationship in the future.

## 5 Conclusion

The findings of this study indicated a transient systemic inflammatory response during the acute phase of the seizures in adults with status epilepticus. However, this response gradually returns to baseline levels following treatment. Unfortunately, there is a lack of robust clinical evidence to establish a clear correlation between the severity of status epilepticus in adults and the systemic inflammatory response.

This study also has some shortcomings. First, it is a retrospective study. Second, the duration and etiology of status epilepticus and their relationships with NLR, PLR, MLR, SII, and PIV were not assessed. Despite these shortcomings, these results still had crucial scientific implications. Future research by this group intends to employ a prospective cohort study approach, which will consider the etiology of status epilepticus and minimize selection bias to elucidate further whether the systemic inflammatory response can predict the severity and outcomes of status epilepticus.

## Data availability statement

The raw data supporting the conclusions of this article will be made available by the authors, without undue reservation.

## Ethics statement

The studies involving humans were approved by the Ethics Committee of Huai’an Third People’s Hospital. The studies were conducted in accordance with the local legislation and institutional requirements. The participants provided their written informed consent to participate in this study. Written informed consent was obtained from the individual(s) for the publication of any potentially identifiable images or data included in this article.

## Author contributions

XS: Data curation, Investigation, Methodology, Writing – original draft. XZ: Data curation, Investigation, Writing – original draft. SS: Data curation, Formal analysis, Writing – original draft. HP: Data curation, Formal analysis, Writing – original draft. CH: Data curation, Formal analysis, Funding acquisition, Writing – original draft. TS: Data curation, Formal analysis, Writing – original draft. SW: Project administration, Supervision, Writing – review & editing. JX: Project administration, Supervision, Writing – review & editing.
